# Endophytic *Berkeleyomyces rouxiae* producing podophyllotoxin and deoxypodophyllotoxin with antibacterial, antioxidant, and cytotoxic activities

**DOI:** 10.3389/fphar.2026.1992195

**Published:** 2026-10-30

**Authors:** Anviksha Anviksha, M. Sudhakara Reddy

**Affiliations:** Department of Biotechnology, Thapar Institute of Engineering and Technology, Patiala, Punjab, India

**Keywords:** biosynthesis, endophytic fungi, natural products, *Podophyllum hexandrum*, secondary metabolites

## Abstract

**Background:**

*Podophyllum hexandrum* is a medicinal plant valued for podophyllotoxin and deoxypodophyllotoxin, whose sustainable production remains challenging. Endophytic fungi represent potential alternative sources of these bioactive metabolites.

**Methods:**

Endophytic fungi isolated from *P. hexandrum* were screened using thin-layer and high-performance liquid chromatography. The promising isolate was characterized using morphological, molecular, chromatographic, and spectroscopic analyses. The antibacterial, antioxidant, and cytotoxic activities of the fungal-derived compounds were evaluated.

**Results:**

Among 25 isolates, PH12 was identified as Berkeleyomyces rouxiae, producing podophyllotoxin (53.8 µg/g DW) and deoxypodophyllotoxin (32.3 µg/g DW), with structural analyses confirming metabolite identity. Fungal podophyllotoxin (fPT) and deoxypodophyllotoxin (fDPT) showed IC50 values of 2.491 ± 0.08 and 2.602 ± 0.09 µg/mL against Escherichia coli, and 2.388 ± 0.07 and 2.528 ± 0.07 µg/mL against Staphylococcus aureus, respectively, compared with 2.467 ± 0.08 and 2.519 ± 0.09 µg/mL for their standards against E. coli and 2.243 ± 0.06 and 2.459 ± 0.08 µg/mL against S. aureus. The fPT and fDPT demonstrated concentration-dependent antioxidant activity, with IC50 values of 325.6 and 404.2 µg/mL in the DPPH assay and 339.3 and 465.1 µg/mL in the reducing power assay, compared with 302.7 and 370.9 µg/mL and 306.6 and 439.1 µg/mL for standards. Cytotoxicity against MCF-7 cells showed IC50 values of 1.336 µg/mL for fPT and 18.32 ng/mL for fDPT, comparable to 1.284 µg/mL and 16.46 ng/mL for standards.

**Conclusion:**

This is among the first reports of B. rouxiae producing both lignans, highlighting its potential as a sustainable source of pharmacologically important compounds with antibacterial, antioxidant, and cytotoxic activities.

## 1 Introduction

The increasing demand for natural compounds with potent therapeutic potential has spurred interest in secondary metabolites derived from plants and their associated endophytic microorganisms. Among these bioactive compounds, lignans such as podophyllotoxin are pharmacologically valuable, with well-documented cytotoxic and antiviral properties (Miranda-Vera et al., 2023; Shao et al., 2026). Podophyllotoxin, a naturally occurring aryl tetralin lignan, serves as a precursor for semi-synthetic anticancer drugs such as etoposide and teniposide, which are extensively used in the treatment of lung cancer, testicular cancer, and lymphomas (Zhang et al., 2020; Shah et al., 2021). However, the complex stereochemistry of podophyllotoxin, featuring four contiguous chiral centers, renders total chemical synthesis economically unviable and technically challenging (Yousefzadi et al., 2010; Pandey, 2026). As a result, current pharmaceutical production relies heavily on natural extraction from plants such as *Podophyllum hexandrum* and *Podophyllum peltatum* (Shah et al., 2021).

Deoxypodophyllotoxin, a structural analog of podophyllotoxin, has also shown significant pharmacological potential. While lacking the C-7 hydroxyl group present in podophyllotoxin, deoxypodophyllotoxin exhibits comparable cytotoxic activity against various cancer cell lines and serves as a direct precursor for the biosynthesis of podophyllotoxin through biotransformation (Jin et al., 2022). Despite its therapeutic value, DPT remains relatively understudied compared to podophyllotoxin, and microbial sources of this compound are particularly scarce in the literature. Simultaneously producing podophyllotoxin and deoxypodophyllotoxin by a single microbial strain would offer distinct advantages for pharmaceutical development, enabling more flexible production strategies for these structurally related lignans.

*Podophyllum hexandrum*, a rare medicinal herb native to the Himalayan regions, contains higher podophyllotoxin yields than its North American counterpart (Motyka et al., 2023). However, due to overharvesting and habitat degradation, the availability of this endangered species is becoming increasingly limited, raising significant concerns about sustainability and ecological impact (Wani et al., 2025). Furthermore, the plants are generally slow-growing and are propagated through seeds, which exhibit a low survival rate (Dogra et al., 2013). These limitations have necessitated the exploration of alternative biological sources that are both renewable and scalable.

One promising approach to circumvent these challenges involves using endophytic fungi as microbial factories for bioactive compound production. Endophytes are symbiotic microorganisms that inhabit the internal tissues of plants without causing harm (Abdalla and McGaw, 2018). These microorganisms are known to participate in complex biochemical interactions with their host plants, including the biosynthesis of secondary metabolites that mimic or even exceed those produced by the host (Wen et al., 2022). Endophytes have already been shown to produce an array of important plant-derived compounds, including taxol, camptothecin, and vincristine, validating their potential as alternative sources of pharmaceutically significant molecules (Gupta et al., 2023).

Previous studies have identified *Aspergillus*, *Fusarium*, and *Alternaria* species capable of producing podophyllotoxin or related lignans, primarily as endophytes of *Juniperus, Dysosma* or *Podophyllum* species (Li, 2007; Kusari et al., 2009; Nguyen et al., 2023; Tan et al., 2026). However, *Berkeleyomyces rouxiae* has not previously been associated with the biosynthesis of podophyllotoxin or deoxypodophyllotoxin. This study aimed to investigate endophytic fungi associated with *P. hexandrum* as potential microbial sources of these lignans and to evaluate their bioactivity. We hypothesized that endophytic fungi inhabiting *P. hexandrum* are capable of producing podophyllotoxin and deoxypodophyllotoxin with measurable antibacterial, antioxidant and cytotoxic activities. Notably, this study reports the novel identification of *Berkeleyomyces rouxiae* as a dual producer of podophyllotoxin and deoxypodophyllotoxin.

## 2 Materials and methods

### 2.1 Collection of *Podophyllum hexandrum*

Fresh, healthy leaf and rhizome segments of *Podophyllum hexandrum* were collected from five individual plants from a natural population in Kullu (Himachal Pradesh) India (32°20′N, 77°21′E; altitude ∼2,000 m). The collected specimens were carefully placed in sterile polyethene bags and transported to the laboratory under cold conditions. A voucher specimen of the plant was prepared and submitted to the Herbarium Museum of Punjabi University, Patiala, Punjab (India) under voucher number 63628 (PUN) for future reference.

### 2.2 Isolation of endophytic fungi from surface-sterilized plant tissues

The collected plant samples were subjected to surface sterilization to eliminate epiphytic microorganisms while preserving internal endophytes. Samples were washed thoroughly under running tap water for 5–10 min, then rinsing in sterile distilled water. Subsequent sterilization involved immersion in 70% ethanol for 1 min, 4% sodium hypochlorite for 3 min, and a final rinse in 70% ethanol for 1 min (Singh et al., 2020). The sterilized segments, of approximately 1 cm length, were washed thrice with sterile distilled water to remove residual sterilants and dried on sterile blotting paper. Segments were cut and inner tissue was aseptically placed onto potato dextrose agar (PDA) plates containing 100 mg/L streptomycin to suppress bacterial growth with four segments being plated on each plate. The sterilized tissue segments were placed in PDA plates without the removal of their surface tissues to test sterilization efficacy and no microbial growth on these plates validated the test. Plates were sealed with parafilm, incubated at 25 °C for up to 7 days, and checked daily (Tan et al., 2018). Hyphal growth emerging from the internal tissues was sub-cultured to fresh PDA plates until pure fungal isolates were obtained.

### 2.3 Morphological and molecular identification of the fungal isolate

The isolated endophytic fungus (PH12) was identified through both morphological and molecular approaches. Microscopic features were examined from slide cultures stained with lactophenol cotton blue and observed under a compound microscope (400×) and the identification was carried out based on hyphal characteristics, conidiogenous structures, and conidial morphology using standard fungal taxonomic keys (Barnet and Hunter, 1998). For molecular analysis, the fungus was cultured on PDA plates overlaid with a cellophane sheet at 25 °C for 10 days to promote mycelial growth. The resulting mycelia were scraped using sterile pipette tips, freeze-dried, and subjected to DNA extraction following the protocol outlined by van Kan et al. (1991).

The internal transcribed spacer (ITS) region of the fungal DNA was amplified using universal fungal primers (White et al., 1990)- ITS1 (5′-TCC​GTA​GGT​GAA​CCT​GCG​G-3′) as the forward primer and ITS4 (5′-TCC​TCC​GCT​TAT​TGA​TAT​GC-3′) as the reverse primer via polymerase chain reaction (PCR) on a GenAmp 9700 thermal cycler (Applied Biosystems, United States). The amplified PCR product was sequenced using Sanger sequencing. The resulting sequence was compared to homologous sequences in the GenBank database of the National Center for Biotechnology Information (NCBI), United States, using the BLASTN algorithm (Altschul et al., 1990). Multiple sequence alignment was performed using ClustalW, and phylogenetic analysis was conducted with MEGA11 software employing the neighbor-joining method to determine the evolutionary relationship of the isolated strain (Kaur et al., 2022). The final sequence was submitted to GenBank, and the accession number was assigned.

### 2.4 Extraction of secondary metabolites (fPT and fDPT)

All fungal isolates were cultured using mycelial discs (5 mm diameter) in 100 mL potato dextrose broth (PDB) in 250 mL Erlenmeyer flasks and incubated at 25 °C under static conditions for 21 days. Each fermentation experiment was performed in triplicate. The fungal cultures were filtered through sterile muslin cloth to separate mycelia from the broth. Both biomass and broth were subjected to solvent extraction using chloroform (stabilized with amylene): methanol (4:1 v/v) (Kour et al., 2008). The organic layer was evaporated under reduced pressure using a rotary evaporator at 35 °C. Crude fungal extracts were analyzed using thin-layer chromatography (TLC) to screen endophytic fungi for the production of podophyllotoxin and deoxypodophyllotoxin. The analysis was performed on silica gel GF254 plates (Sigma-Aldrich, St. Louis, United States) using a chloroform (stabilized with amylene): methanol solvent system in a 24:1 ratio. Standard podophyllotoxin (sPT) and standard deoxypodophyllotoxin (sDPT) (Sigma-Aldrich, St. Louis, United States) were used as a reference, and spots were visualized under UV light at 254 nm. For fungi testing positive for metabolite production, crude extracts from the culture filtrate and fungal mycelium were combined to determine the total fungal podophyllotoxin (fPT) and fungal deoxypodophyllotoxin (fDPT) content.

### 2.5 Column chromatography and high-performance liquid chromatography (HPLC) analysis

Crude extract (100 mg) was adsorbed onto silica gel (60–120 mesh) and dry-loaded onto a pre-equilibrated silica column (45 cm × 2.5 cm column packed with silica up to the 30 cm mark). Elution was carried out with a gradient of chloroform (stabilized with amylene): methanol (9.5:0.5, 9:1, 8.5:1.5, 8:2, 7:3 and 6:4 v/v), with 150 mL of each solvent mixture. Fractions (10 mL each) were collected (yielding a total of 90 fractions) and dried to get the powdered samples, and TLC confirmed the fractions showing presence of podophyllotoxin or deoxypodophyllotoxin.

HPLC analysis was performed using a Shimadzu HPLC LC-20AD system with a photodiode array (PDA) detector and a Shim-pack GIST C18 (Shimadzu, Japan) reverse-phase column (250 × 4.6 mm, 5 µm). The mobile phase consisted of methanol: water (62:38 v/v) in isocratic mode, with a flow rate of 1.0 mL/min (Sharma et al., 2018). Detection was performed at 290 nm and the injection volume was 20 µL. Purified fractions were dissolved in methanol, sonicated for 10 min, and filtered through a 0.22 µm Millipore syringe filter. sPT and sDPT were prepared in concentrations ranging from 20–100 μg/mL to construct a calibration curve. Peak areas were plotted against concentrations to generate a regression equation (R^2^ > 0.99) for quantifying fPT and fDPT in the samples.

### 2.6 Structural characterization using NMR and HRMS

#### 2.6.1 UV-vis and FTIR spectroscopy

The UV absorption spectrum was recorded using a Cary 60 UV-Vis spectrophotometer (Agilent, United States) over a 200–400 nm wavelength range. For analysis, 1 mg of fPT or fDPT was dissolved in 1 mL of methanol and used for measurement. The obtained spectra were compared with those of the respective standards for confirmation. Additionally, the fungal sample was analyzed using FTIR spectroscopy to verify the presence of expected functional groups. The FTIR spectra were obtained using a Shimadzu IRTracer-100 spectrophotometer (Shimadzu, Kyoto, Japan) equipped with a diamond attenuated total reflectance (ATR) accessory, a KBr beam splitter, a DTGS detector, and OPUS software. For analysis, 2 mg of fPT or fDPT was thoroughly mixed with 200 mg of KBr to form pellets. Scans were conducted at a resolution of 4 cm^−1^, with 100 scans collected over a wavenumber range of 4000–400 cm^−1^. The spectra were then compared to the reference FTIR spectrum of sPT and sDPT to confirm structural features.

#### 2.6.2 NMR spectroscopy

Samples (50 mg) were dissolved in approximately 0.5 mL CDCl_3_ and analyzed using a 400 MHz instrument (Jeol, United States). The ^1^H and ^13^C NMR spectra were recorded at 400 and 100 MHz, respectively, and chemical shifts(δ) were expressed in ppm relative to tetramethylsilane (TMS) as the internal standard. The resulting NMR spectra were cross-referenced with existing podophyllotoxin and deoxypodophyllotoxin data to validate their structure.

#### 2.6.3 High-resolution mass spectrometry (HRMS)

High-resolution mass spectrometry (HRMS) was used to confirm the molecular weight and structure of the isolated compound. Analysis was performed using electrospray ionization in positive-ion mode (ESI+) on a Waters Xevo G2-XS QTOF mass spectrometer coupled with UPLC. The mass spectrum was acquired over an m/z range of approximately 100–1200 with a collision energy of 30 V. The mass spectra were acquired in the positive-ion mode to determine the molecular ions and fragmentation patterns of podophyllotoxin and deoxypodophyllotoxin.

### 2.7 Antibacterial assay

The antimicrobial activity of fPT and fDPT was evaluated against *Escherichia coli* (Gram-negative) and *Staphylococcus aureus* (Gram-positive) using broth microdilution coupled with an MTT-based colorimetric viability assay, adapted from Ye et al. (2013) and Requena et al. (2019). Bacterial colonies were suspended in sterile saline (0.85% NaCl) and adjusted to a 0.5 McFarland standard and subsequently diluted to approximately 10^5^ CFU/mL. Fungal extract, standard PT, and DPT were prepared at 10 mg/mL in DMSO (1% v/v final concentration) and serially diluted in LB broth. Equal volumes (100 µL) of each dilution and bacterial suspension were added to sterile 96-well plates. The concentrations reported for the antibacterial assay represent the final concentrations in the wells after mixing equal volumes of sample dilution and bacterial suspension (0, 0.3125, 0.625, 1.25, 2.5, 5, 7.5, and 10 μg/mL). Growth, sterility, DMSO solvent, and streptomycin-treated positive controls were included. The growth control contained bacteria without test compound, the sterility control contained medium without bacteria, the DMSO control contained bacteria with the corresponding DMSO concentration, and streptomycin served as the positive control. Plates were incubated at 37 °C for 24 h, followed by addition of 10 µL MTT solution (5 mg/mL in PBS) and incubation for a further 4 h. Cell viability was assessed based on MTT reduction to purple formazan, with optical density measured at 570 nm. Inhibition (%) was calculated relative to the growth control. The MIC was defined as the lowest concentration at which no visible purple color development was observed compared with the growth control. For determination of the minimum bactericidal concentration (MBC), aliquots from the MIC and higher concentration wells showing no visible color change were inoculated onto LB agar plates and incubated at 37 °C for 24 h. The MBC was defined as the lowest concentration showing no bacterial growth on the agar plates. All assays were performed in triplicate.

### 2.8 Antioxidant activity assays

#### 2.8.1 DPPH radical scavenging assay

The DPPH assay was employed to evaluate the antioxidant activity of the fPT and fDPT, following the method outlined by Borkotoky et al. (2024). A 100 µM methanolic stock solution of the DPPH radical was prepared. Equal volumes of DPPH solution and methanolic solutions of test samples at final concentrations of 0, 50, 100, 200, 300, 500 and 1000 μg/mL were mixed and incubated in the dark at room temperature for 30 min. Absorbance was then recorded at 517 nm. The antioxidant activity of the samples was determined by calculating the percentage inhibition of DPPH radicals. The IC_50_ value was determined, representing the concentration required to scavenge 50% of the DPPH radicals. Gallic acid (Sigma-Aldrich, St. Louis, United States) served as the positive control, while the negative control consisted of a mixture of methanol and DPPH without any sample or drug. DPPH radical scavenging activity was evaluated based on the decrease in absorbance following reaction with the test samples. Scavenging activity was expressed as the percentage reduction in absorbance relative to the negative control. The IC_50_ values were determined from the concentration-response curves.

#### 2.8.2 Reducing power assay

The reducing power of fPT and fDPT was evaluated to determine their potential as an electron donor, following the method described by Saeed et al. (2012). Extracted samples at final concentrations of 0, 50, 100, 200, 300, 500 and 1,000 μg/mL and gallic acid (used as a positive control) were mixed with phosphate buffer (0.2 M, pH 6.6, 500 µL) and potassium ferricyanide solution (1% w/v, 500 µL). A mixture without any sample served as the negative control. The reaction mixtures were incubated at 50 °C for 2 minutes, after which 250 µL of trichloroacetic acid (TCA, 10% w/v) was added. The mixtures were then centrifuged at 6,000 rpm for 8 minutes. Equal volumes of the resulting supernatants and one-fifth volume of 0.1% ferric chloride were added to deionized water. Following a 10-min incubation, absorbance was recorded at 700 nm. The test solution’s yellow color shifted to greenish blue depending on its reducing capacity. Higher absorbance values at increased concentrations indicated stronger reducing power. Reducing power was expressed relative to the negative control based on the absorbance of the sample and control. IC_50_ values were determined from the concentration-response curves.

### 2.9 Cytotoxicity evaluation against MCF-7 cells

MCF-7 human breast cancer cells (RRID: CVCL_0031) were procured from the National Centre for Cell Science (India) and cultured in Minimum Essential Medium with Earle’s salts, L-Glutamine, NEAA and 2.2 g per litre Sodium bicarbonate (HiMedia, India) supplemented with 10% fetal bovine serum (FBS), 100 IU/mL penicillin, and 100 μg/mL streptomycin. Cells were maintained at 37 °C in a humidified 5% CO_2_ atmosphere. To study the cytotoxic effect, cells were seeded in 96-well plates containing growth medium and incubated overnight to allow adherence. Following this, cells were treated with varying concentrations of fPT (0, 0.625, 1.25, 2.5, 5, 10, 20, and 40 μM; equivalent to 0, 0.259, 0.518, 1.036, 2.072, 4.144, 8.288, and 16.576 μg/mL) and fDPT (0, 2.5, 5, 10, 20, 40, 80, and 160 nM; equivalent to 0, 1.00, 1.99, 3.98, 7.97, 15.94, 31.87, and 63.74 ng/mL), dissolved in dimethyl sulfoxide (DMSO; final concentration 0.1%), and incubated at 37 °C in a humidified atmosphere containing 5% CO_2_ for 48 h. After incubation, cells were washed with phosphate-buffered saline (PBS) to remove residual media. Untreated cells served as a control. Subsequently, 10 µL of MTT solution (5 mg/mL; 3-(4,5-dimethylthiazol-2-yl)-2,5-diphenyltetrazolium bromide) was added to each well, and the plates were incubated for an additional 4 h. The supernatant was removed, and 100 µL of DMSO was added to solubilize the resulting formazan crystals. Absorbance was measured at 570 nm (Chu et al., 2014) and cell viability was expressed as a percentage relative to the untreated control. The IC_50_ values were determined from the resulting dose-response curves.

### 2.10 Data analysis

The IC_50_ values were calculated from dose-response curves using nonlinear regression analysis in GraphPad Prism (version 8). For antibacterial, antioxidant and cytotoxicity assays, concentration of the test compounds was treated as the independent variable, and the corresponding response (percentage inhibition or cell viability) was considered the dependent variable. Statistical significance among treatment groups was evaluated using one-way analysis of variance (ANOVA), followed by Tukey’s *post hoc* test for multiple comparisons. A p-value of < 0.05 was considered statistically significant.

## 3 Results

### 3.1 Isolation of endophytic fungi from *Podophyllum hexandrum*

Fungal colonies with distinct morphologies were successfully isolated from the surface-sterilized tissues of *P. hexandrum*. A total of 60 tissue segments were processed, from which fungal growth was observed in 68.33% of the segments. This resulted in 25 unique fungal isolates (PH1-PH25) from rhizome and leaf tissues. Of these, 15 isolates were recovered from rhizome tissues and 10 from leaf tissues, indicating a higher colonization frequency in the rhizome. Isolate PH12, which was subsequently selected for further study, was recovered from the rhizome tissue. These isolates were maintained on PDA plates for subsequent evaluation. The crude extracts of all isolates were subjected to TLC to detect podophyllotoxin-related metabolites (Supplementary Figure S1). sPT and sDPT exhibited retention factors (Rf) of 0.39 and 0.42, respectively. Among the screened isolates, one isolate, PH12, showed positive TLC bands corresponding to podophyllotoxin and deoxypodophyllotoxin and was selected for subsequent studies.

### 3.2 Identification of the endophyte

The fungal isolate PH12 formed colonies on PDA with dark-centred, cottony aerial mycelium and a spreading growth pattern that gradually became olivaceous to black with age (Figures 1A,B). Microscopic examination revealed septate, branched hyphae with phialidic conidiogenous cells producing hyaline, ellipsoidal to oblong conidia (Figures 1C,D). ITS rDNA sequencing produced a fragment of ∼600 bp. BLAST analysis showed 99.1% similarity to *Berkeleyomyces rouxiae*. Phylogenetic analysis (Figure 1E) clustered PH12 firmly within the *B. rouxiae* clade with strong bootstrap support, confirming its identity. The ITS sequence was deposited in GenBank under accession number PX136385. The fungal strain PH12 has been deposited at the Microbial Type Culture Collection and Gene Bank (MTCC), Institute of Microbial Technology (IMTECH), Chandigarh, India, under accession number MTCC 14069.

**FIGURE 1:**
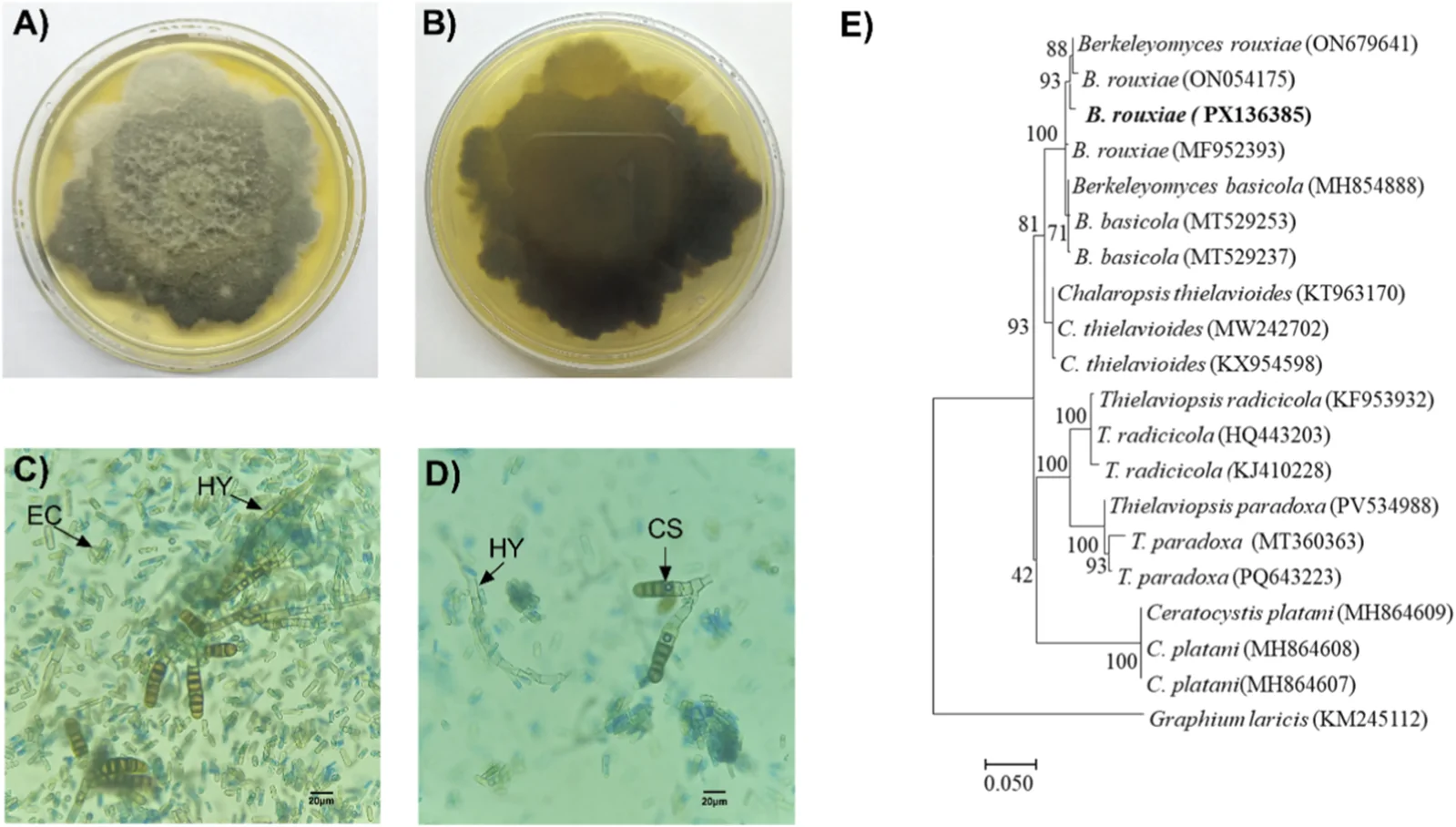
**(A)** Dorsal and **(B)** ventral view of the fungal isolate PH12 on Potato Dextrose Agar plate **(C–D)** microscopic images of the isolate at ×400 magnification showing endoconidia (EC), hyphae (HY) and conidiospore (CS). **(E)** Phylogenetic tree showing resemblance of the isolated fungus with *Berkeleyomyces rouxiae*. Bar represents 5 substitutions per 100 nucleotide position. Numbers at branch nodes refer to bootstrap values based on 1,000 replicates.

### 3.3 Purification and quantification of podophyllotoxin and deoxypodophyllotoxin

The crude extract of PH12 was subjected to silica gel column chromatography and the fractions positive for fPT and fDPT in TLC (Supplementary Figures S2A,B) were pooled and analyzed using HPLC for metabolite identification and yield determination (Supplementary Figure S2C). HPLC analysis revealed distinct peaks corresponding to podophyllotoxin and deoxypodophyllotoxin. The retention time of sPT was 11.237 min (Figure 2A).

**FIGURE 2:**
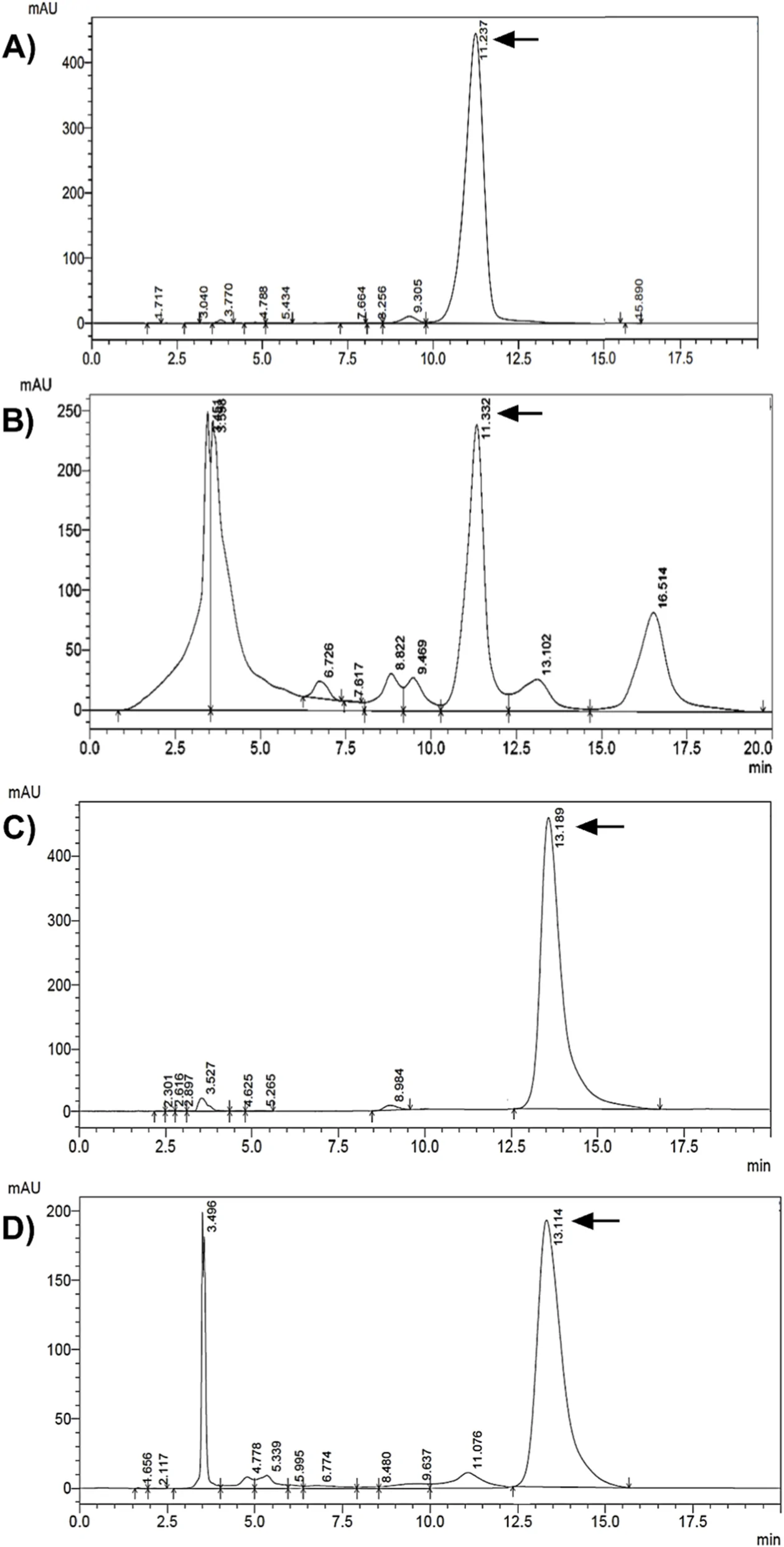
High-performance liquid chromatography (HPLC) profiles of podophyllotoxin and deoxypodophyllotoxin. **(A)** Standard podophyllotoxin (sPT, 11.237 min). **(B)** Fungal podophyllotoxin (fPT, 11.332 min). **(C)** Standard deoxypodophyllotoxin (sDPT, 13.189 min). **(D)** Fungal deoxypodophyllotoxin (fDPT, 13.114 min).

In comparison, fPT appeared at 11.332 min (Figure 2B). Similarly, sDPT showed a retention time of 13.189 min (Figure 2C), and fDPT eluted at 13.114 min (Figure 2D). Calibration curves of standards (R^2^ > 0.99) enabled quantification. The combined yield was estimated at 53.8 μg/g DW for fPT and 32.3 μg/g DW for fDPT. Based on preliminary extraction profiles, the majority of the metabolites were associated with the culture filtrate, contributing approximately 39.42 μg/g DW (fPT) and 24.64 μg/g DW (fDPT), while the mycelia accounted for roughly 14.38 μg/g DW (fPT) and 7.68 μg/g DW (fDPT). Isolate PH12 produced 2.31 ± 0.21 g dry biomass/100 mL culture, corresponding to 23.1 ± 2.1 g/L. Based on the metabolite yields normalized to dry biomass, the corresponding volumetric yields were approximately 1.24 mg/L for fPT and 0.75 mg/L for fDPT.

### 3.4 Characterization of purified compounds

#### 3.4.1 UV-vis and FTIR spectroscopy

The UV absorption spectra of fPT (Figure 3A) and fDPT (Figure 3B) exhibited a strong peak at 290 nm, closely resembling their respective standards. FTIR analysis of fPT (Figure 3C) showed characteristic peaks at 3308 cm^−1^ (–OH stretching), 2945 cm^−1^ (-CH stretching in benzene ring), 1657 cm^−1^ (C=O stretch in lactone ring), 1450–1413 cm^−1^ (aromatic C=C vibrations), and 1114–1018 cm^−1^ (C-O-C stretching). In contrast, fDPT (Figure 3D) displayed similar absorption features with minor variations in intensity but lacked the -OH stretching peak.

**FIGURE 3:**
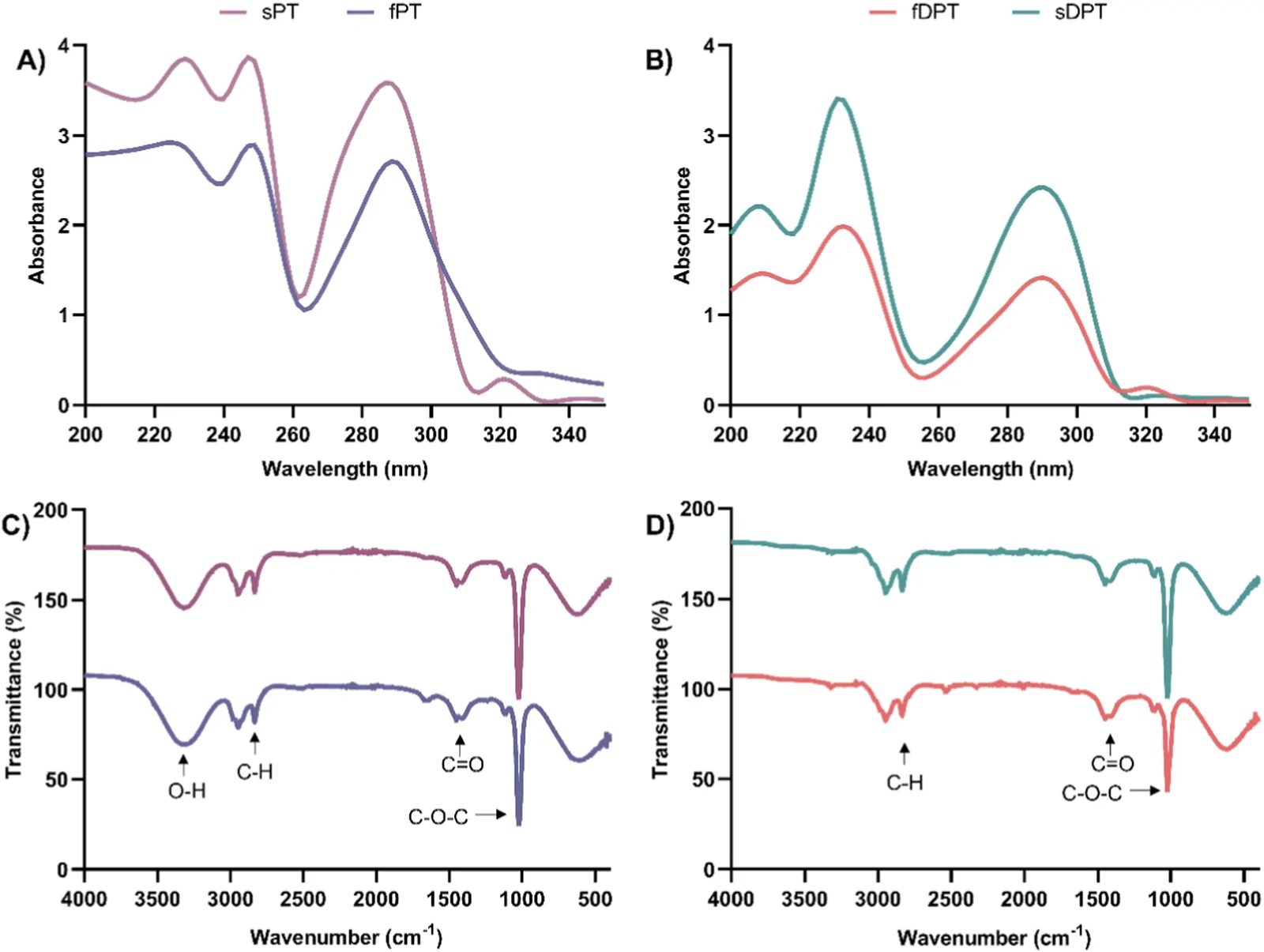
Spectroscopic characterization of podophyllotoxin and deoxypodophyllotoxin. UV-Vis spectra of **(A)** sPT and fPT, and **(B)** sDPT and fDPT. FTIR spectra of **(C)** sPT and fPT, and **(D)** sDPT and fDPT.

#### 3.4.2 NMR spectroscopy

The NMR analyses confirmed the structural identities of both metabolites. fPT exhibited distinctive ^1^H NMR resonances (Supplementary Figure S3) at 7.11 (s, 1H), 6.50 (s, 1H), 6.37 (s, 2H), 5.99 (d, *J* = 7.4 Hz, 1H), 5.97 (d, *J* = 6.8 Hz, 1H), 4.77 (d, *J* = 6.4 Hz, 1H), 4.57 (d, *J* = 4.9 Hz, 2H), 4.06 (d, *J* = 5.8 Hz, 1H), 3.79 (s, 3H), 3.74 (s, 6H), 2.88 - 2.73 (m, 2H), 2.46 (d, *J* = 7.7 Hz, 1H) and ^13^C NMR peaks (Supplementary Figure S4) at δ 174.66 (C-9), 152.77 (C-3/C-5), 147.83 (C-4′), 146.73 (C-3′), 137.22 (C-4), 135.65 (C-1), 133.45 (C-6′), 131.25 (C-1′), 109.72 (C-5′), 108.51 (C-2/C-6), 106.40 (C-2′), 101.62 (-OCH_2_O-), 72.80 (C-7′), 71.55 (C-9′), 60.88 (4-OMe), 56.44 (3,5-OMe), 45.41 (C-8), 44.21 (C-7), 40.83 (C-8′). fDPT exhibited additional distinguishing ^1^H NMR resonances (Supplementary Figure S5) at δ 6.65 (s, 1H), 6.51 (s, 1H), 6.35 (s, 2H), 5.94 (d, 1H), 5.91 (t, *J* = 7.9 Hz, 1H), 4.59 (d, *J* = 6.6 Hz, 1H), 4.46 – 4.39 (m, *J* = 4.9 Hz, 1H), 3.91 (d, *J* = 6.9 Hz, 1H), 3.84 (s, 3H), 3.73 (s, 6H), 3.07 (d, *J* = 8.7 Hz, 1H), 2.75 (d, *J* = 7.6 Hz, 1H), 2.72 (t, *J* = 10.5 Hz, 2H) and ^13^C NMR signals (Supplementary Figure S6) at δ 174.84 (C-9′), 152.52 (C-3′/C-5′), 147.11 (C-4′), 146.80 (C-3′), 137.33 (C-4), 136.22 (C-1), 130.72 (C-6), 128.31 (C-1′), 110.53 (C-5), 108.50 (C-2′/C-6′), 101.22 (-OCH_2_O-), 72.01 (C-9), 60.84 (4-OMe), 56.33 (3,5-OMe), 47.50 (C-7′), 43.83 (C-7), 33.13 (C-8), 32.82 (C-8′). Minor additional signals were observed, likely due to trace impurities or residual solvents. The ^1^H and ^13^C NMR spectral data of the isolated compounds were compared with previously reported data for authentic podophyllotoxin and deoxypodophyllotoxin. The observed chemical shifts and signal patterns were in close agreement with literature values, confirming the structural identity of the compounds (Ikeda et al., 1998; Lazzarotto et al., 2019).

#### 3.4.3 HRMS analysis

High-resolution mass spectrometry confirmed the identities of fPT and fDPT. fPT (Figure 4A) displayed a protonated ion peak at m/z 415.1391 [M + H] ^+^, with a fragment at m/z 397.1286 [loss of H_2_O] ^+^. Spectrum of fDPT (Figure 4B) showed a molecular ion at m/z 399.1395 [M + H] ^+^, and fragmentation produced a major ion at m/z 231.0654, consistent with the loss of a trimethoxybenzene. Further fragmentation of this ion generated m/z 213.0501 and 187.0749, corresponding to losses of H_2_O and CO_2_, respectively. The m/z 213.0501 ion subsequently yielded m/z 185.0729 through loss of CO, whereas m/z 187.0749 yielded m/z 157.0682 through loss of formaldehyde, followed by formation of m/z 129.0701 through loss of CO.

**FIGURE 4:**
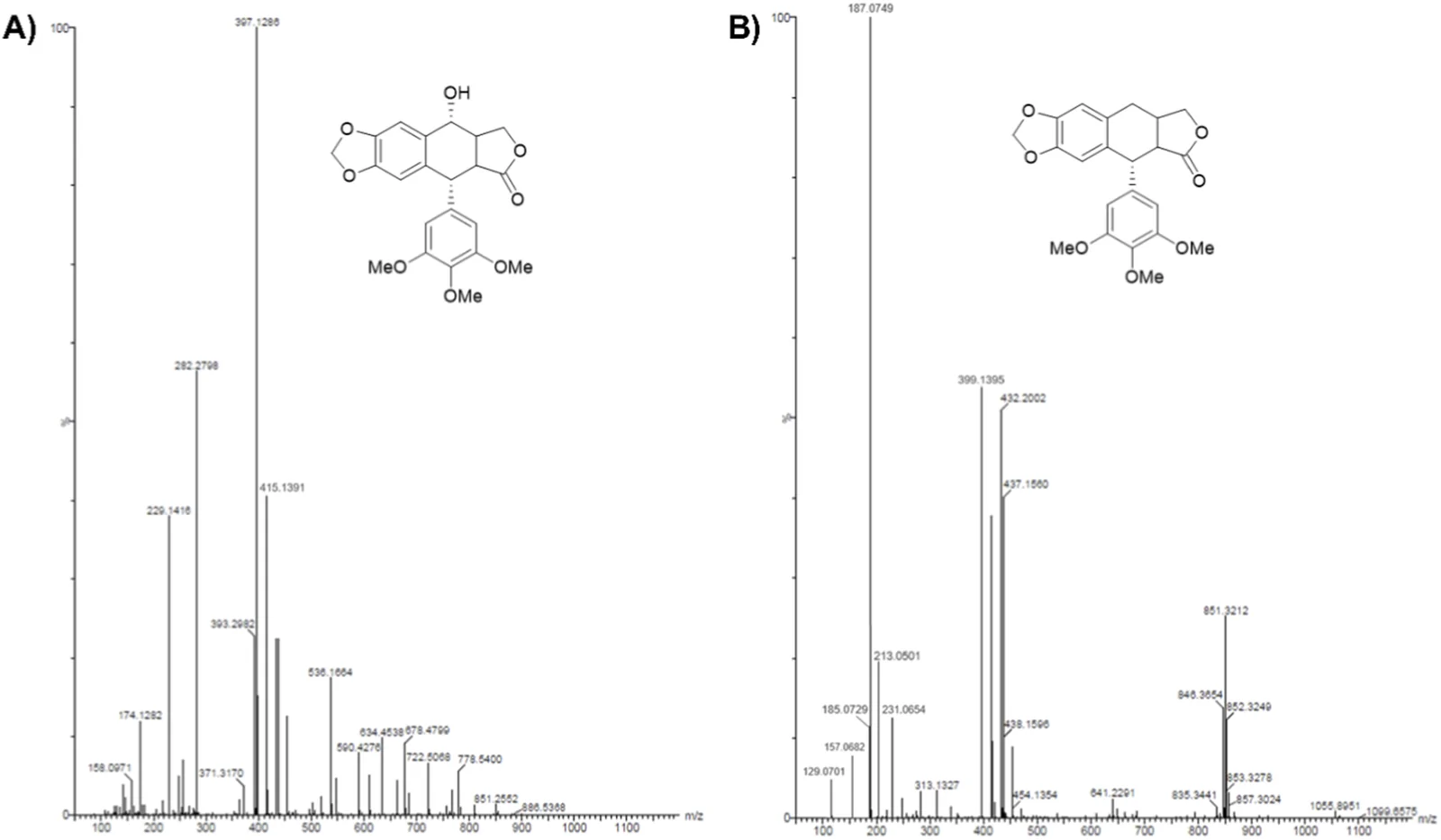
High-resolution mass spectrometry (HRMS) analysis of fungal compounds. **(A)** fPT showing protonated molecular ion peak at m/z 415.1391 [M + H]+and fragment ion at m/z 397.1286. **(B)** fDPT showing protonated molecular ion peak at m/z 399.1395 [M + H]+with characteristic fragments at m/z 231.0654, 213.0501, 187.0749, 185.0729, 157.0682, and 129.0701.

In addition to the molecular ion peaks, minor signals corresponding to adduct formation and low-intensity background ions were observed. The observed molecular ion peaks and fragmentation patterns were consistent with those reported for podophyllotoxin and deoxypodophyllotoxin, providing additional support for their identities (Kusari et al., 2009; Ivanova et al., 2023). The proposed fragmentation pathways of PT and DPT based on the observed HRMS fragment ions are presented in Figure 5.

**FIGURE 5:**
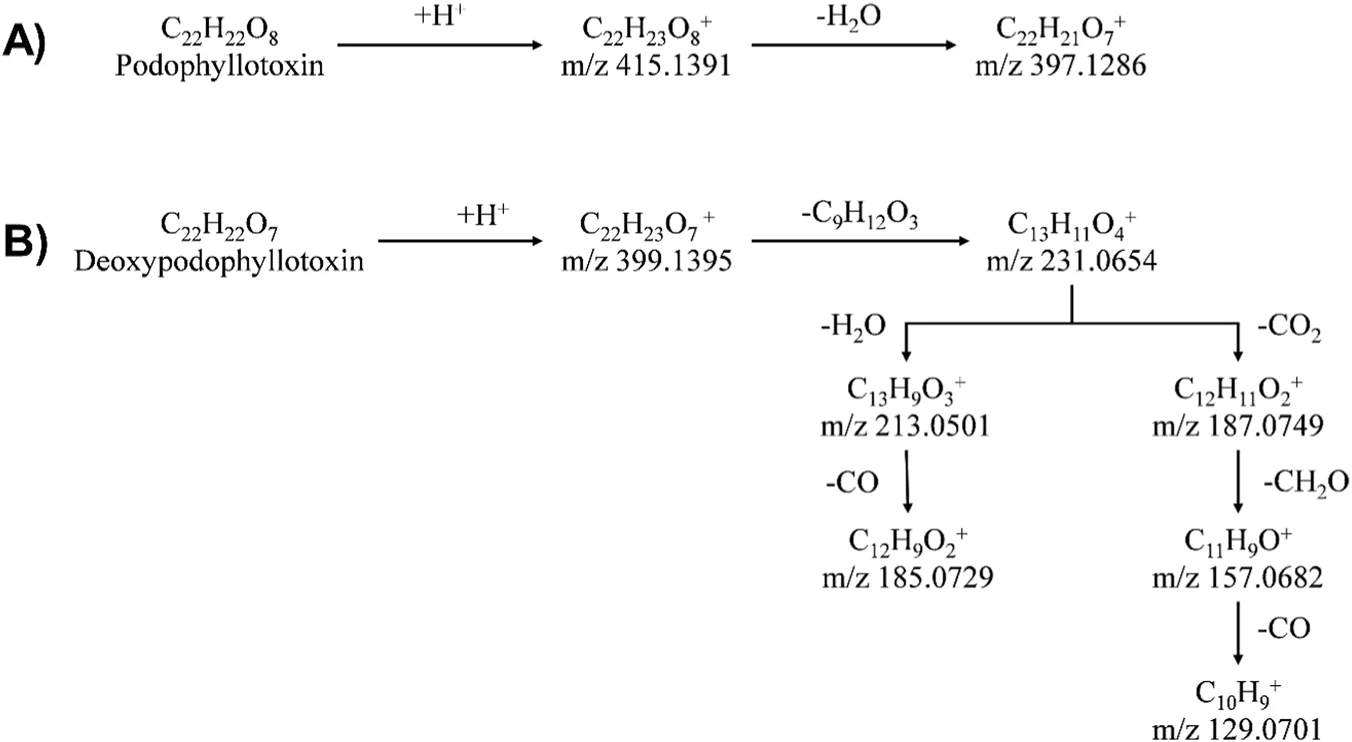
Proposed fragmentation patterns of podophyllotoxin **(A)** and deoxypodophyllotoxin **(B)** based on the observed HRMS fragment ions.

### 3.5 Antibacterial assay

The antimicrobial activity of PT and DPT samples was evaluated against *Escherichia coli* and *Staphylococcus aureus* based on their IC_50_ values. The fPT exhibited IC_50_ values of 2.491 ± 0.08 μg/mL against *E. coli* and 2.388 ± 0.07 μg/mL against *S. aureus* (Figures 6A,B). The fDPT showed comparatively higher IC_50_ values of 2.602 ± 0.09 and 2.528 ± 0.07 μg/mL against *E. coli* and *S. aureus*, respectively. Standard PT showed IC_50_ values of 2.467 ± 0.08 μg/mL (*E. coli*) and 2.243 ± 0.06 μg/mL (*S. aureus*), while standard DPT exhibited values of 2.519 ± 0.09 and 2.459 ± 0.08 μg/mL, respectively (Figures 6A,B). In comparison, streptomycin demonstrated substantially lower IC_50_ values of 0.425 ± 0.03 μg/mL against *E. coli* and 0.799 ± 0.03 μg/mL against *S. aureus* (Figure 6C), indicating greater antimicrobial potency than the PT and DPT samples. For *S. aureus*, PT showed an MIC of 8 μg/mL and an MBC of 16 μg/mL, while DPT showed an MIC and MBC of 16 μg/mL. Against *E. coli*, PT showed an MIC and MBC of 16 μg/mL, whereas DPT showed an MIC of 16 μg/mL and an MBC of 32 μg/mL.

**FIGURE 6:**
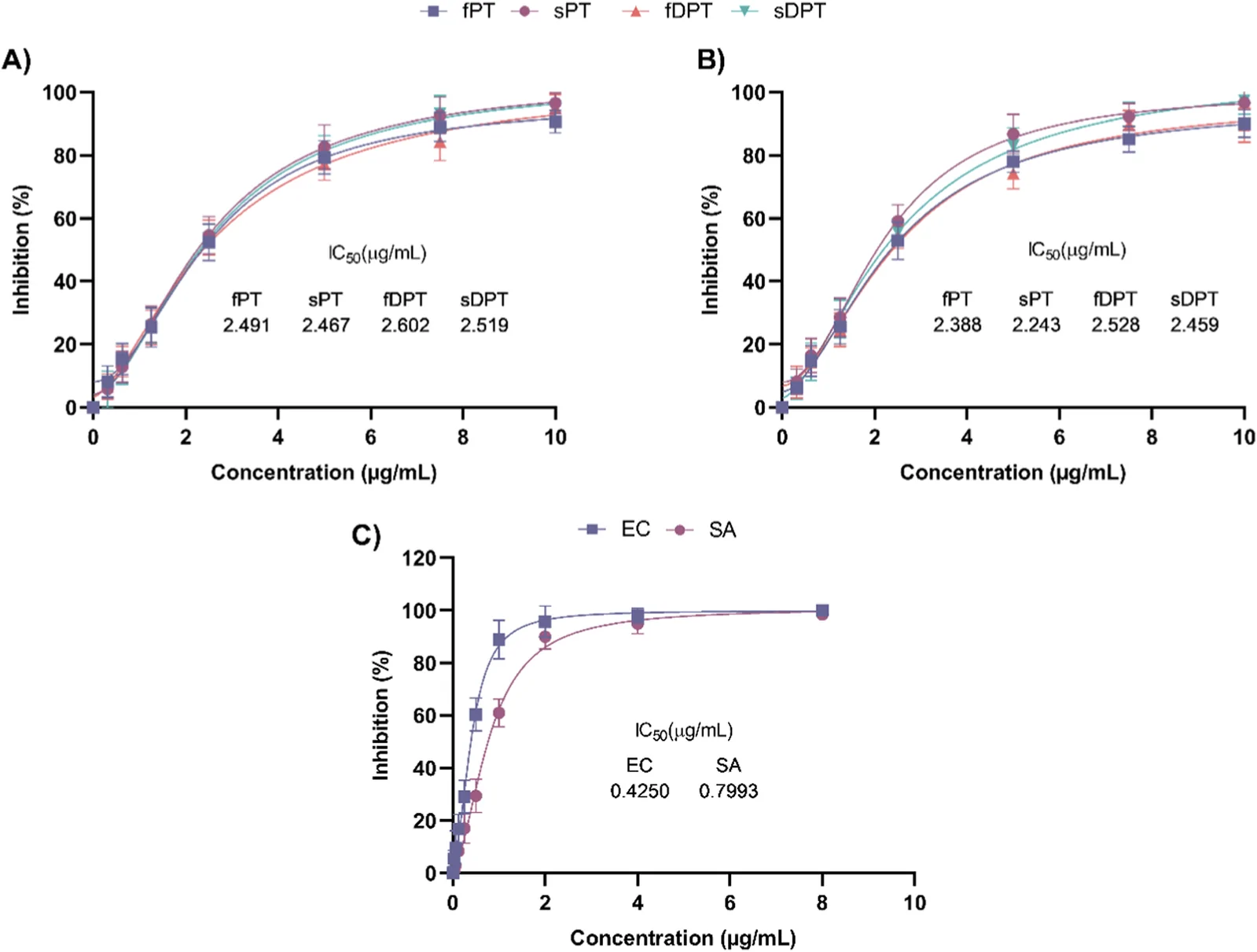
Antibacterial activity of fungal podophyllotoxin (fPT) and fungal deoxypodophyllotoxin (fDPT) compared with their respective standards (sPT and sDPT), with streptomycin as the positive control. **(A)** Antibacterial activity of fPT, sPT, fDPT, and sDPT against *Escherichia coli*. **(B)** Antibacterial activity of the same compounds against *Staphylococcus aureus*. **(C)** Antibacterial activity of streptomycin against *E. coli* (EC) and *S. aureus* (SA). Error bars represent mean ± standard deviation (n = 3).

### 3.6 Antioxidant activity assays

#### 3.6.1 DPPH radical scavenging activity

Fungal podophyllotoxin and deoxypodophyllotoxin both demonstrated concentration-dependent DPPH scavenging activity (Figure 7A). The IC_50_ values were 325.6 μg/mL (fPT) and 404.2 μg/mL (fDPT), comparable to sPT (302.7 μg/mL) and sDPT (370.9 μg/mL). Gallic acid exhibited much stronger activity (IC_50_ = 65.77 μg/mL).

**FIGURE 7:**
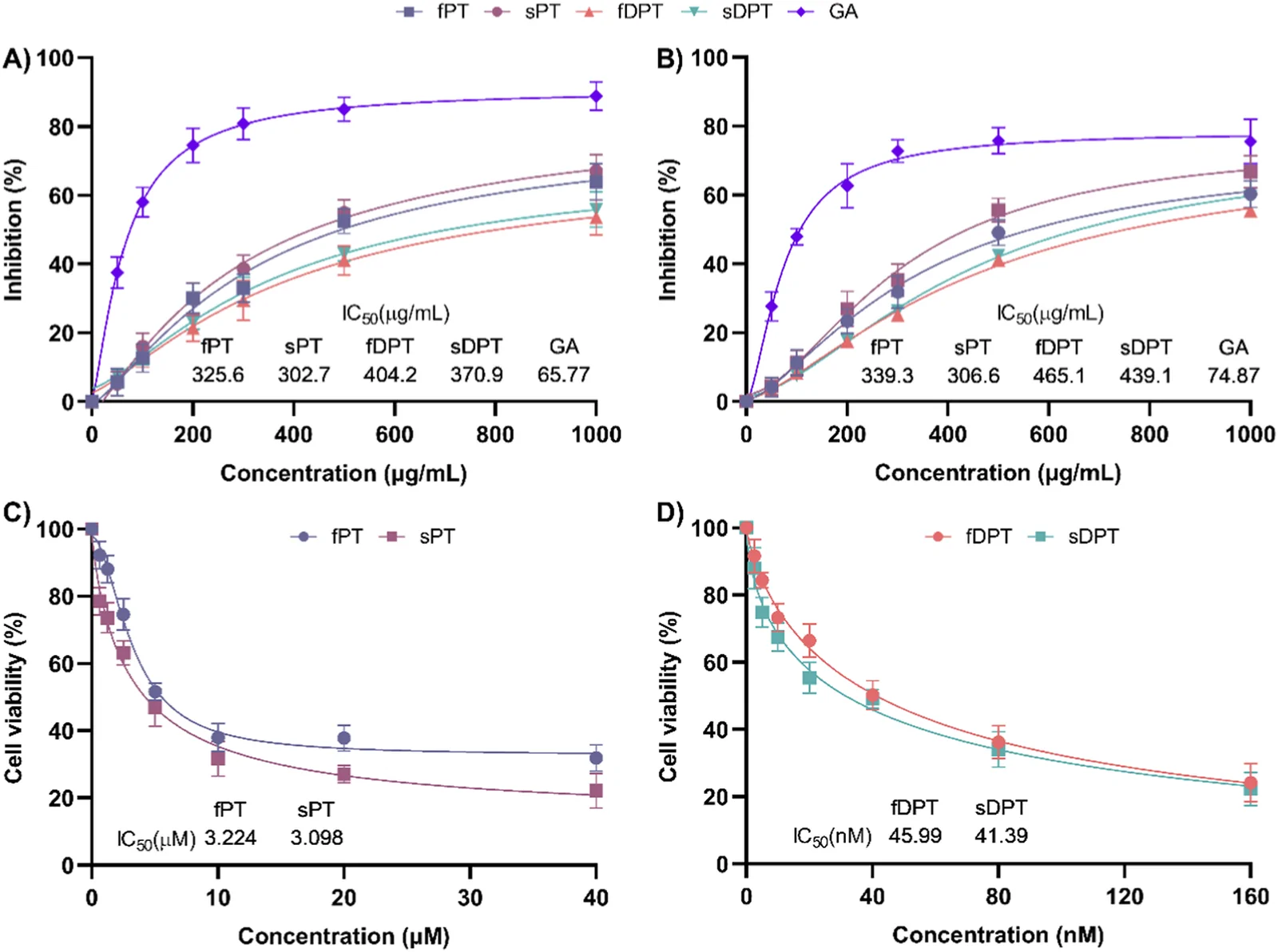
Bioactivity of fungal podophyllotoxin (fPT) and deoxypodophyllotoxin (fDPT) compared with standards. **(A)** DPPH radical scavenging activity showing concentration-dependent inhibition and IC_50_ values. **(B)** Reducing power assay indicating electron-donating capacity. **(C)** Cytotoxicity of sPT and fPT against MCF-7 breast cancer cells, as determined by the MTT assay. **(D)** Cytotoxicity of sDPT and fDPT against MCF-7 cells. Error bars represent mean ± standard deviation (n = 3). Gallic acid (GA) served as the positive control for antioxidant assays.

#### 3.6.2 Reducing power assay

The reducing capacity of both compounds increased with concentration, confirming their electron-donating potential (Figure 7B). IC_50_ values were calculated as 339.3 μg/mL for fPT and 465.1 μg/mL for fDPT. Standards showed IC_50_ values of 306.6 μg/mL (sPT) and 439.1 μg/mL (sDPT), while gallic acid demonstrated superior activity (IC_50_ = 74.87 μg/mL).

### 3.7 Cytotoxicity assay against MCF-7 cells

The MTT assays revealed strong antiproliferative activity of both fungal-derived metabolites (Figures 7C,D). fPT inhibited MCF-7 growth with an IC_50_ of 1.336 μg/mL, while fDPT showed an IC_50_ of 18.32 ng/mL sPT and sDPT exhibited comparable potencies (IC_50_ values of 1.284 μg/mL and 16.46 ng/mL, respectively). These results confirmed that fPT and fDPT retain strong cytotoxic effects, comparable to their standard counterparts.

## 4 Discussion

This study aimed to investigate endophytic fungi associated with *Podophyllum hexandrum* as potential microbial sources of podophyllotoxin and deoxypodophyllotoxin, and to evaluate the bioactivity of the metabolites produced. Among the 25 isolates obtained, strain PH12 was identified as *Berkeleyomyces rouxiae* and demonstrated the ability to produce both podophyllotoxin and deoxypodophyllotoxin. The podophyllotoxin yield from *B. rouxiae* was calculated as 53.8 μg/g DW, placing it on par with other fungal endophytes reported in the literature. Comparable yields have been described in *Trametes hirsuta* from *P. hexandrum* (30 μg/g DW (Puri et al., 2006)), *Fusarium oxysporum* from *Juniperus recurva* (28 μg/g DW (Kour et al., 2008)), and *Fusarium solani* from *P. hexandrum* (29 μg/g DW (Nadeem et al., 2012)). *Mucor fragilis* from *Sinopodophyllum hexandrum* achieved a similar yield of 49.3 μg/g DW (Huang et al., 2014). At the higher end of the productivity spectrum, elicitation strategies significantly increased yields, for instance, *Fusarium proliferatum* from *Dysosma versipellis* produced 314 μg/g DW PT when supplemented with host extracts or phenylalanine (Nguyen et al., 2023). Thus, while the yield obtained from *B. rouxiae* is moderate, its ability to produce both podophyllotoxin and deoxypodophyllotoxin naturally distinguishes it from previously reported isolates.

The structural identity of the fungal-derived metabolites was supported by a combination of spectroscopic analyses, including UV-Vis, FTIR, NMR, and HRMS. The UV absorption maxima around 290 nm were consistent with the characteristic chromophore of aryltetralin lignans such as podophyllotoxin and deoxypodophyllotoxin (Dall’Acqua et al., 2006; Salih et al., 2021). The FTIR spectra further confirmed the presence of key functional groups, including lactone carbonyl and aromatic moieties, in agreement with standard compounds (Coates, 2006). The ^1^H and ^13^C NMR data showed strong concordance with previously reported values for these lignans, particularly in the aromatic, methoxy, and oxygenated carbon regions, supporting structural assignment (Ikeda et al., 1998; Lazzarotto et al., 2019). The HRMS analysis provided additional confirmation through molecular ion peaks and fragmentation patterns consistent with the expected molecular weights (Kusari et al., 2009; Ivanova et al., 2023). Similar multi-technique approaches have been employed in earlier reports of fungal-derived podophyllotoxin and related lignans, reinforcing the reliability of the present identification. Collectively, these data confirm that the isolated metabolites correspond to podophyllotoxin and deoxypodophyllotoxin.

Reports of deoxypodophyllotoxin biosynthesis in endophytes are comparatively rare. *Aspergillus fumigatus* from *Juniperus communis* was shown to produce DPT at ∼100 μg/g DW (Kusari et al., 2009), representing one of the few detailed accounts. In our study, *B. rouxiae* produced DPT at 32.3 μg/g DW, somewhat lower than *A. fumigatus*. Importantly, this is the first report of a single endophyte producing both podophyllotoxin and deoxypodophyllotoxin simultaneously. Such dual production is particularly valuable, as podophyllotoxin and deoxypodophyllotoxin represent sequential steps in the lignan biosynthetic pathway. This suggests that *B. rouxiae* may possess a more complete enzymatic machinery for lignan biosynthesis, offering a unique microbial platform with enhanced flexibility for pharmaceutical applications.

The consistent antibacterial activity observed for podophyllotoxin and deoxypodophyllotoxin supports its reproducible bioactivity and potential suitability for microbial production systems (Kusari et al., 2012; Gouda et al., 2016). Podophyllotoxin and deoxypodophyllotoxin showed activity against both Gram-positive (*S. aureus*) and Gram-negative (*E. coli*) bacteria, indicating broad-spectrum antibacterial potential. This activity is consistent with reported mechanisms of lignans involving disruption of bacterial membrane integrity and interference with intracellular targets such as DNA and topoisomerases (Saleem et al., 2010; Cushnie and Lamb, 2011).

Both podophyllotoxin and deoxypodophyllotoxin displayed radical-scavenging and electron-donating properties, though weaker than gallic acid. These results are consistent with prior studies showing that podophyllotoxin derivatives generally exhibit moderate antioxidant activity, attributable to hydroxyl and methoxy substitutions on the aryltetralin scaffold (Amarowicz et al., 2004; Dar et al., 2017). Deoxypodophyllotoxin, which lacks the free hydroxyl group at C-4, demonstrated weaker antioxidant potential than podophyllotoxin in our assays. This observation suggests that the presence and position of free hydroxyl groups play an important role in enhancing radical scavenging and electron-donating activity.

The potent cytotoxic effects of both podophyllotoxin and deoxypodophyllotoxin against MCF-7 cells are consistent with their well-established antineoplastic mechanisms of microtubule destabilization and topoisomerase II inhibition. Podophyllotoxin is known to bind tubulin at the colchicine site, impeding microtubule polymerization. At the same time, structurally related derivatives, including deoxypodophyllotoxin, are reported to inhibit topoisomerase II, leading to DNA damage and apoptosis (Zhao et al., 2015). The IC_50_ values for fPT were comparable to those of the respective standards, indicating that the fungal-derived compound retains significant biological activity. This retention of cytotoxic potency underscores the fidelity of the fungal metabolic machinery in producing bioactive compounds. Podophyllotoxin exhibited potent cytotoxicity across diverse cancer cell types. For instance, in leukemia models using *Juniperus* extracts, it showed IC_50_ values as low as 0.0040 μg/mL against J45.01 cells and 0.0286 μg/mL against CEM/C1 cells (Och et al., 2015). Additionally, its mechanism includes topoisomerase II inhibition and mitotic arrest, causing double-stranded DNA breaks and cellular apoptosis (Anviksha and Reddy, 2025).

Deoxypodophyllotoxin demonstrated exceptionally potent cytotoxicity at nanomolar concentrations. These results are aligned with reports where deoxypodophyllotoxin isolated from *Dysosma versipellis* and *Anthriscus sylvestris* demonstrated potent nanomolar cytotoxicity against breast and esophageal cancer cells (Khaled et al., 2017; Kwak et al., 2020).

From an ecological perspective, the ability of *B. rouxiae* to synthesize podophyllotoxin and deoxypodophyllotoxin illustrates the phenomenon of metabolic mimicry, where endophytes reproduce or extend the specialized metabolite repertoire of their host plants (Gouda et al., 2016; Tiwari and Bae, 2022). Such chemistry may contribute to mutualistic interactions, providing chemical defence to fungus and host. From a conservation standpoint, this is highly significant: *P. hexandrum* is slow-growing, endangered, and listed under CITES Appendix II, making large-scale plant harvesting unsustainable (Chaurasia et al., 2012). Endophyte-based production thus provides a promising sustainable route to meet the growing pharmaceutical demand for podophyllotoxin and deoxypodophyllotoxin.

## 5 Conclusions and future perspectives

This study establishes *Berkeleyomyces rouxiae* as a previously unreported endophyte capable of producing both podophyllotoxin and deoxypodophyllotoxin. The identities of these lignans were confirmed through chromatographic and spectroscopic analyses, and their bioactivities were comparable to standard compounds. The dual production of these pharmaceutically important metabolites highlights the biosynthetic potential of this endophyte. The observed cytotoxicity against MCF-7 cells provides preliminary evidence of their biological activity; however, as cytotoxicity was evaluated using only a single cancer cell line, cancer-cell selectivity and safety toward normal cells could not be assessed. These findings support the exploration of endophytic fungi as sustainable alternatives to plant-derived sources. Future work should focus on fermentation optimization, elicitor screening, genomic pathway elucidation, and *in vivo* pharmacological evaluations. Such efforts could pave the way for sustainable microbial production of these valuable lignans, reducing reliance on endangered *Podophyllum* species while opening avenues for novel drug development.

## Data Availability

The data presented in this study can be found in the GenBank repository under accession number PX136385.
