# Supplementary material for: Endophytic Berkeleyomyces rouxiae producing podophyllotoxin and deoxypodophyllotoxin with antibacterial, antioxidant, and cytotoxic activities
Source: Front Pharmacol. 2026 Oct 30;17:1992195. doi: 10.3389/fphar.2026.1992195 (PMC13759817; doi:10.3389/fphar.2026.1992195)
Supplement: Supplementary file 1 [file Supplementaryfile1.docx]

**Endophytic *Berkeleyomyces rouxiae* Producing Podophyllotoxin and Deoxypodophyllotoxin with Antibacterial, Antioxidant, and Cytotoxic Activities**

**Anviksha Anviksha, M. Sudhakara Reddy^*^**

Department of Biotechnology, Thapar Institute of Engineering and Technology, Patiala, Punjab, 147004, India

**Correspondence**

***Dr. M. Sudhakara Reddy***

[msreddy@thapar.edu](mailto:msreddy@thapar.edu)

**Figure S1** Thin-layer chromatography (TLC) analysis of crude extracts from endophytic fungal isolates (PH1–PH25). Plates (A–E) represent groups of five isolates each: (A) PH1–PH5, (B) PH6–PH10, (C) PH11–PH15, (D) PH16–PH20, and (E) PH21–PH25. Each plate includes reference standards podophyllotoxin (PT) and deoxypodophyllotoxin (DPT).

**Figure S2** (A) Thin-layer chromatogram of fPT, and (B) of fDPT, showing separation of standard (s), culture filtrate extract (f), and mycelial extract (m) under UV light at 254 nm. Standard podophyllotoxin (sPT) and deoxypodophyllotoxin (sDPT) were used as references. (C) Yield of podophyllotoxin (fPT) and deoxypodophyllotoxin (fDPT) obtained from chromatographic fractions (F1-F4).

**Figure S3** ^1^H NMR spectrum of fungal podophyllotoxin dissolved in deuterated chloroform (CDCl_3_).

**Figure S4** ^13^C NMR spectrum of fungal podophyllotoxin dissolved in deuterated chloroform (CDCl_3_).

**Figure S5** ^1^H NMR spectrum of fungal deoxypodophyllotoxin dissolved in deuterated chloroform (CDCl_3_).

**Figure S6** ^13^C NMR spectrum of fungal deoxypodophyllotoxin dissolved in deuterated chloroform (CDCl_3_).
